# Risk factors and a nomogram model for recurrence of benign paroxysmal positional vertigo: a multicenter cross-sectional study

**DOI:** 10.3389/fneur.2025.1542090

**Published:** 2025-04-03

**Authors:** Qingchun Pan, Bei Li, Kai Zou, Jing Zhang, Yuanling Wang, Xiaoming Tang

**Affiliations:** Department of Otolaryngology Head and Neck Surgery, Affiliated Hospital of North Sichuan Medical College, Nanchong, China

**Keywords:** recurrence, benign paroxysmal positional vertigo, a nomogram, prediction model, risk factor

## Abstract

**Objective:**

To explore factors influencing the recurrence of benign paroxysmal positional vertigo (BPPV) within 1 year after treatment with canalith repositioning procedure (CRP) and to construct a risk prediction model with a nomogram. This model aims to offer a scientific basis for of high-risk groups’ early identification and to support the development of prevention strategies.

**Methods:**

Data of BPPV patients diagnosed and successfully treated with CRP of 5 hospitals in Sichuan Province from Jan 2020 to Mar 2024 were retrospectively analyzed. The patients were divided into a training set of BPPV and a set of validation with a 7:3 ratio. After organizing the clinical data, the training set’s patients were arranged into recurrent and non-recurrent subgroups based on the recurrence of BPPV within one-year post-treatment. Factors affecting BPPV recurrence were found using LASSO regression; then, multivariate logistic regression (MLR) analysis was used to build a nomogram-style risk prediction model. The “receiver operating characteristic” (ROC) curve, Hosmer-Lemeshow calibration curve, clinical “decision curve analysis” (DCA), and clinical impact curve were employed to assess the model’s discrimination, accuracy, and clinical applicability.

**Results:**

600 patients in all were involved; 180 were in the validation set and 420 in the training set, following a 7:3 split. The overall recurrence rate of BPPV within 1 year was 39.17% (235/600 cases). The recurrence rates in the validation and training sets were 39.44% (71/180 cases) and 39.05% (164/420 cases), respectively. LASSO regression and logistic regression analyses identified hypertension, hyperglycemia, migraine, HADS-A, and 25(OH)D as significant factors for recurrence. On the basis of these factors, a nomogram prediction model for recurrence was established. The validation and training sets had an area under the ROC curve of 0.723 (95%CI: 0.645–0.801) and 0.728 (95%CI: 0.679–0.777), respectively. The “Hosmer-Lemeshow goodness-of-fit (HLGOF) test” indicated satisfactory calibration (training set: chi-square = 8.708, *p* = 0.368; validation set: chi-square = 13.303, *p* = 0.102). The analysis exhibited an excellent consistency between the model’s predicted probabilities and actual outcomes. DCA and clinical impact curve analyses indicated a positive net clinical benefit at various threshold probability levels, affirming the clinical value of the nomogram model in forecasting BPPV recurrence.

**Conclusion:**

BPPV patients treated with CRP exhibit a high recurrence rate within one-year post-treatment. Hypertension, hyperglycemia, migraine, HADS-A, and 25(OH)D levels were related with increased recurrence risk. The risk prediction model, presented as a nomogram, demonstrated good discrimination and calibration, effectively predicting the BPPV’s recurrence risk within 1 year and offering significant clinical utility.

## 1 Introduction

Benign paroxysmal positional vertigo (BPPV) is among the most prevalent reasons for vertigo in emergency departments, neurology clinics, and otorhinolaryngology outpatient settings, accounting for 34.25% of all cases of dizziness-related disorders. The reported one-year prevalence of BPPV is 1.6%, with a life-time occurrence of 2.4% (1). Clinically, BPPV presents as transient vertigo that occurs suddenly with head position changes and is frequently escorted by nausea, vomiting, and other symptoms (2). Research has indicated that persons with BPPV are at higher risk for subsequent strokes, falls, fractures, hypertension, dementia, and psychological distress (3). Consequently, BPPV significantly reduces patients’ quality of life, impairs daily functioning, and increases societal burden.

The “Canalith repositioning procedure” (CRP) is an effective treatment for BPPV patients, which cures most cases. However, some BPPV patients experience relapse after CRP treatment (4). Research indicates that up to 80% of BPPV patients experience a recurrence within 1 year of treatment, with a recurrence rate of 50% over 10 years (5, 6). Although BPPV is self-limiting and generally responds well to treatment, its recurrent nature affects 68% of patients’ work performance, leads 4% of patients to change jobs, and results in 6% quitting employment. Recurrent attacks can also lead to depression, anxiety, and mental health issues (7, 8). Thus, studying BPPV recurrence risk factors is essential to reduce medical costs and alleviate patient distress.

Zhu et al. reported that patients of BPPV with migraine, Meniere’s disease, hyperlipidemia, and hypertension are at a higher recurrence risk (9). A meta-analysis of 14 studies including 3,060 BPPV patients reported that female sex, hyperlipidemia, hypertension, vitamin D deficiency diabetes, and osteoporosis are significant risk factors for recurrence of BPPV. Nevertheless, the influence of other potential risk factors, including advanced head trauma, Meniere’s disease, advanced age, and migraine requires further investigation (5). Contrarily, Fu et al. identified poor physical activity and prolonged decubitus as independent risk factors for BPPV recurrence, while age, Meniere’s disease, sudden deafness, migraine, hypertension, hyperlipidemia, diabetes, and mental factors did not significantly impact recurrence risk (10). Thus, research on BPPV recurrence risk factors has largely focused on variables such as sex, age, hypertension, hyperglycemia, and vitamin D deficiency, but no consensus exists across domestic and international studies. Additionally, few studies have constructed predictive models for BPPV recurrence, and current research on risk factors is often limited to single-factor, single-center studies or lists of influencing factors lacking external validation and clear risk thresholds.

A risk prediction model, incorporating multiple variables, predicts outcomes, aiding clinicians in assessing patients’ disease risks and prognoses. Various models have been widely applied in clinical settings, enhancing prevention, diagnosis, and treatment strategies. Some researchers have constructed BPPV onset and residual dizziness risk prediction models using nomograms, which have demonstrated substantial clinical value. Given the high recurrence rate of BPPV, identifying its risk factors could allow high-risk patients’ early identification and facilitate preventive measures to reduce recurrence. However, few predictive models for BPPV recurrence have been developed. Therefore, this multi-center study used the “least absolute shrinkage and selection operator” (LASSO) regression to screen variables, followed by MLR analysis to construct a recurrence nomogram for BPPV patients within 1 year after successful reduction treatment, providing a reference for clinical prevention of BPPV recurrence.

## 2 Materials and methods

### 2.1 Patients and procedures

BPPV patients who were diagnosed and successfully treated with CRP of 5 hospitals in Sichuan Province (Affiliated Hospital of North Sichuan Medical College,Suining Central Hospital,Meishan City People’s Hospital,Nanbu County People’s Hospital) between Jan, 2020 and Mar, 2024 were selected as study participants. Patient data collected included gender, age, years of education, handedness, place of residence, body mass index, smoking status, alcohol use, daily exercise, and comorbidities (such as hypertension, hyperlipidemia, hyperglycemia, migraine, cervical spondylosis, Meniere’s disease, osteoporosis, and history of head trauma). Additionally, data on the otolith site, Dizziness Handicap Inventory (DHI) score, “Hospital Anxiety and Depression Scale-Anxiety” (HADS-A) and Depression (HADS-D) scores, “Pittsburgh Sleep Quality Index” (PSQI), serum calcium concentration, and 25-hydroxyvitamin D (25(OH)D) concentration were recorded.

### 2.2 Inclusion criteria

Patients were enrolled based on a criteria as follows: (1) Diagnosis confirmed by established BPPV diagnostic criteria; (2) Successful CRP treatment; (3) Education level > 6 years; (4) Signed informed consent; (5) Vertigo induced by head position changes; (6) Presence of typical positional nystagmus of BPPV; (7) Absence of other central nervous system diseases, vestibular dysfunction, gynecological tumors, thyroid disease, or recent hormone therapy; (8) Successful BPPV diagnosis and CRP treatment; (9) No drug use after successful CRP treatment (1).

### 2.3 Exclusion criteria

Patients were excepted due to the reasons as follows: (1) Unresolved craniocerebral injury; (2) Unresolved suppurative otitis media; (3) Unresolved central nervous system injury or other factors affecting brain structure and function; (4) Mental illness or neurological conditions impeding cooperation; (5) Previous vitamin D, calcium therapy, or osteoporosis treatment (2).

### 2.4 Sample size and allocation

Based on the requirement of 5–10 times the number of independent variables for model construction and accounting for a 20% attrition rate, 600 patients were ultimately included. With 24 independent variables, patients were randomly allocated in a 7:3 ratio, resulting in 420 patients for training and 180 patients for validation. This study was approved by the Ethics Committee of the AHNSMC (Ethics Number: 2020ER035-1), and all participants gave informed consent for examination and treatment.

### 2.5 Treatment and follow-up

Dislocation tests, such as the “Dix-Hallpike and Roll-Test,” were conducted using an infrared video electronystagmogram (Denmark, International Hearing, VO425). The nystagmus duration and latency were seen and recorded, and bilateral slow-phase angular velocity was measured. Diagnostic criteria followed those outlined by the Otolaryngology Branch of the Chinese Medical Association in 2017 (3).

### 2.6 BPPV CRP treatment

The following CRP treatments were administered based on semicircular canal involvement: (1) Epley maneuver for patients with posterior semicircular canal BPPV; (2) Yacovino maneuver for anterior semicircular canal BPPV; (3) Combined Epley and Barbecue maneuvers for multiple semicircular canal BPPV.

Following diagnosis and treatment, all patients were monitored through phone or outpatient follow-ups conducted by the same physician for 1 year. During this period, recurrence of episodic positional vertigo and positive repositioning test results were recorded to identify recurrences.

### 2.7 Measurement indexes

#### 2.7.1 Evaluation of CRP efficacy

Based on the 2017 BPPV diagnosis and treatment criteria, according to patients’ postural vertigo and nystagmus reactions, the efficacy of CRP was classified as follows: (1) Recovery: Complete disappearance of vertigo symptoms; (2) Improvement: Alleviation of vertigo and/or nystagmus, though not entirely eliminated; (3) Ineffective: No relief in dizziness or nystagmus, or symptoms have worsened (4).

#### 2.7.2 Hypertension

The 2010 Hypertension Guidelines defined hypertension as a diastolic blood pressure more than 90 mmHg or a systolic blood pressure more than 140 mmHg (11).

#### 2.7.3 Hyperlipidemia

Hyperlipidemia was diagnosed with a fasting serum total cholesterol level of ≥5.72 mmol/L and a triglyceride level of ≥1.7 mmol/L (12).

#### 2.7.4 Hyperglycemia

Two hours after a meal, a fasting blood glucose level of ≥7.0 mmol/L and/or a blood glucose level of ≥11.1 mmol/L characterized as hyperglycemia (13).

#### 2.7.5 Bone density

A Hologic dual-energy X-ray absorptiometer assessed the lumbar vertebrae’s (L1–L4) bone mineral density (BMD). BMD T-scores were calculated as follows: (1) Normal: T value > −1.0; (2) Decreased bone mass: T value −1.0 to −2.5; (3) Osteoporosis: T value < −2.5 (14).

#### 2.7.6 Blood calcium and 25(OH)D concentrations

After confirming and successfully repositioning BPPV, venous blood from the elbow was collected, centrifuged, and analyzed. 25(OH)D concentration was measured using an immunofluorescence analysis system, and blood calcium concentration was measured using a semi-automatic analyzer. Criteria for 25(OH)D were as follows: (1) Sufficiency: 20–100 ng/mL. (2) Deficiency: <12 ng/mL; (3) Insufficiency: 12–20 ng/mL.

Both deficiency and insufficiency indicated an abnormal 25(OH)D level (15). Normal blood calcium levels were defined as 2.25–2.75 mmol/L, with values below 2.25 mmol/L considered abnormal (16).

#### 2.7.7 Dizziness handicap inventory (DHI) score

The DHI score was used to quantitatively assess dizziness severity: (1) Mild impairment: 0 to 30 points; (2) Moderate impairment: 31 to 60 points; (3) Severe impairment: 61 to 100 points (17).

#### 2.7.8 Hospital anxiety and depression scale (HADS)

The HADS was employed to assess depression and anxiety, comprising 14 items (each rated 0–3, with 8 items scored in reverse). HADS includes both HADS-D and HADS-A subscales, each scored out of 21 points. The scoring standards were as follows: (1) Normal: 0–7; (2) Mild abnormality: 8–10; (3) Abnormal: ≥11.

Patients with mild or abnormal scores on either HADS-A or HADS-D were defined as having anxiety or depression, respectively (18).

#### 2.7.9 PSQI

The PSQI was utilized to evaluate sleep quality. A total score of 0–7 indicated “good” sleep quality, and a score ≥ 8 indicated “poor” sleep quality (19).

### 2.8 Data analysis

Data analysis was done via statistical programing. Means ± standard deviation (±s) for measurement data with a normal or very normal distribution were calculated; an independent sample t-test was then used to compare the two groups. Using the rank sum test for group comparisons, data not typically distributed were stated as median (interquartile range). Categorical data were stated as frequency or percentage; the φ^2^ test was used for comparisons.

In a 7:3 ratio, patients were paired randomly to training and validation sets. Utilizing relapse as the outcome variable, LASSO regression was utilized to screen independent variables. LASSO regression added a penalty term to the least squares method to compress the estimated parameters, identifying factors with a significant impact on the dependent variable (20). Variables for model fitting were determined based on LASSO regression results and analyzed using multivariable logistic regression to build a nomogram prediction model.

Plotting the “receiver operating characteristic” (ROC) curve, the “area under the curve” (AUC) was computed to evaluate model predictive effectiveness using values nearer to 1 indicating increased prediction accuracy. Calibrating the Hosmer-Lemeshow test produced a calibration curve that assessed the goodness of fit of the model. Setting a test level of *α* = 0.05, *p* = 0.05 was deemed statistically significant.

## 3 Results

### 3.1 Demographic and clinical information in the validation and training sets

600 patients in all were randomly allocated to the training set (*n* = 420) and validation set (*n* = 180) at a 7:3 ratio. Patients in both sets were evaluated based on age, sex, years of education, handedness, place of residence, body mass index, smoking status, alcohol use, daily exercise, and comorbid conditions (including hypertension, hyperlipidemia, hyperglycemia, migraine, cervical spondylosis, Meniere’s disease, osteoporosis, and history of head trauma). No significant variances were observed among the groups in relations of otolith location, DHI score, HADS-A, HADS-D, PSQI, VAS, blood calcium concentration, or 25(OH)D concentration (*p* > 0.05). Table 1 presents the general information for the validation and training sets.

**Table 1:** Demographic and clinical information in training set and validation set [±s or M (P25, P75)].

	Training set (*n* = 420)	Validation set (*n* = 180)	*X*^2^	*p*
Age (years)			0.343	0.558
<60	327 (77.86)	144 (80.00)		
≥60	93 (22.14)	36 (20.00)		
Gender (%)			2.679	0.102
Male	177 (42.14)	63 (35.00)		
Female	243 (57.86)	117 (65.00)		
Education years (years)			2.106	0.147
<9	269 (64.05)	104 (57.78)		
≥9	151 (35.95)	76 (42.22)		
Dextromanuality (%)			–	–
Left	420 (100)	180 (100)		
Right	0 (0)	0 (0)		
Place of abode (%)			0.039	0.843
Country	120 (28.57)	50 (27.78)		
City	300 (71.43)	130 (72.22)		
BMI (%)			0.137	0.934
<24 kg/m2	64 (15.24)	28 (15.56)		
24-28 kg/m2	200 (47.62)	88 (48.89)		
≥28 kg/m2	156 (37.14)	64 (35.56)		
Stroke (%)			0.331	0.565
No	332 (79.05)	146 (81.11)		
Yes	88 (20.95)	34 (18.89)		
Intemperance (%)			0.952	0.329
No	390 (92.86)	171 (95.00)		
Yes	30 (7.14)	9 (5.00)		
Hypertension (%)			3.505	0.061
No	305 (72.62)	117 (65.00)		
Yes	115 (27.38)	63 (35.00)		
Hyperlipemia (%)			0.007	0.936
No	309 (73.57)	133 (73.89)		
Yes	111 (26.43)	47 (26.11)		
Diabetes (%)			0.893	0.345
No	285 (67.86)	115 (63.89)		
Yes	135 (32.14)	65 (36.11)		
Migraine (%)			0.945	0.331
No	299 (71.19)	121 (67.22)		
Yes	121 (28.81)	59 (32.78)		
Cervical spondylosis (%)			0.414	0.520
No	191 (45.48)	87 (48.33)		
Yes	229 (54.52)	93 (51.67)		
Meniere disease (%)			1.345	0.246
No	369 (87.86)	164 (91.11)		
Yes	51 (12.14)	16 (8.89)		
Osteoporosis (%)			0.879	0.348
No	237 (56.43)	109 (60.56)		
Yes	183 (43.57)	71 (39.44)		
History of head trauma (%)			0.008	0.93
No	402 (95.71)	172 (95.56)		
Yes	18 (4.29)	8 (4.44)		
Daily exercise >1 h/day (%)			0.011	0.915
≤1 h/d	205 (48.81)	87 (48.33)		
>1 h/d	215 (51.19)	93 (51.67)		
Sites			3.896	0.273
Posterior semicircular canal	230 (54.76)	107 (59.44)		
Horizontal semicircular canal	99 (23.57)	32 (17.78)		
Anterior semicircular canal	82 (19.52)	34 (18.89)		
Mixed type	9 (2.14)	7 (3.89)		
DHI score (%)			2.51	0.285
Mild	165 (39.29)	69 (38.33)		
Moderate	203 (48.33)	96 (53.33)		
Severe	52 (12.38)	15 (8.33)		
HADS-A scores			0.249	0.618
Normal	129 (30.71)	59 (32.78)		
Abnormal	291 (69.29)	121 (67.22)		
HADS-D scores			0.088	0.766
Normal	377 (89.76)	163 (90.56)		
Abnormal	43 (10.24)	17 (9.44)		
PSQI scores			0.032	0.859
Normal	218 (51.9)	92 (51.11)		
Abnormal	202 (48.1)	88 (48.89)		
Serum calcium (mmol/L)(%)			1.467	0.226
Normal	181 (43.1)	68 (37.78)		
Abnormal	239 (56.9)	112 (62.22)		
25(OH)D (ng/ml) (%)			2.181	0.140
Normal	140 (33.33)	49 (27.22)		
Abnormal	280 (66.67)	131 (72.78)		

### 3.2 Univariate analyses of risk factors

600 patients participated in this research; of these, 235 experienced recurrence within 1 year after manual reduction, while 365 did not, resulting in a recurrence rate of 39.17%. In the training set, comprising 420 patients, 164 recurred and 256 did not, with a recurrence rate of 39.05%. In the verification set of 180 patients, 71 relapsed and 109 did not, with a recurrence rate of 39.44%. Statistically significant differences (< 0.05) were noted between the relapse and non-recurrence groups in hypertension, hyperlipidemia, hyperglycemia, migraine, cervical spondylosis, osteoporosis, HDS-A, HADS-D, PSQI, blood calcium concentration, and 25(OH)D concentration. Table 2 presents the clinical data for the relapsing and non-relapsing groups in the training concentration.

**Table 2:** Demographic and clinical information in training set [±s or M (P25,P75)].

	Non-recurrence group (*n* = 256)	Recurrent group (*n* = 164)	*X*^2^	*p*
Age (years)			1.876	0.171
<60	205 (80.08)	122 (74.39)		
≥60	51 (19.92)	42 (25.61)		
Gender (%)			1.945	0.163
Male	101 (39.45)	76 (46.34)		
Female	155 (60.55)	88 (53.66)		
Education years (years)			1.070	0.301
<9	159 (62.11)	110 (67.07)		
≥9	97 (37.89)	54 (32.93)		
Dextromanuality (%)			–	–
Left	256 (100)	164 (100)		
Right	0 (0)	0 (0)		
Place of abode (%)			0.169	0.681
Country	75 (29.3)	45 (27.44)		
City	181 (70.7)	119 (72.56)		
BMI (%)			0.096	0.953
<24 kg/m2	40 (15.63)	24 (14.63)		
24-28 kg/m2	122 (47.66)	78 (47.56)		
≥28 kg/m2	94 (36.72)	62 (37.8)		
Stroke (%)			1.299	0.254
No	207 (80.86)	125 (76.22)		
Yes	49 (19.14)	39 (23.78)		
Intemperance (%)			2.770	0.096
No	242 (94.53)	148 (90.24)		
Yes	14 (5.47)	16 (9.76)		
Hypertension (%)			14.703	<0.001
No	203 (79.30)	102 (62.20)		
Yes	53 (20.70)	62 (37.80)		
Hyperlipemia (%)			1.116	0.291
No	193 (75.39)	116 (70.73)		
Yes	63 (24.61)	48 (29.27)		
Diabetes (%)			10.717	0.001
No	189 (73.83)	96 (58.54)		
Yes	67 (26.17)	68 (41.46)		
Migraine (%)			21.006	<0.001
No	203 (79.3)	96 (58.54)		
Yes	53 (20.7)	68 (41.46)		
Cervical spondylosis (%)			7.441	0.006
No	130 (50.78)	61 (37.2)		
Yes	126 (49.22)	103 (62.8)		
Meniere disease (%)			0.408	0.523
No	227 (88.67)	142 (86.59)		
Yes	29 (11.33)	22 (13.41)		
Osteoporosis (%)			4.523	0.033
No	155 (60.55)	82 (50.00)		
Yes	101 (39.45)	82 (50.00)		
History of head trauma (%)			0.258	0.611
No	244 (95.31)	158 (96.34)		
Yes	12 (4.69)	6 (3.66)		
Daily exercise >1 h/day (%)			0.036	0.849
≤1 h/d	124 (48.44)	81 (49.39)		
>1 h/d	132 (51.56)	83 (50.61)		
Sites			0.456	0.928
Posterior semicircular canal	142 (55.47)	88 (53.66)		
Horizontal semicircular canal	58 (22.66)	41 (25.00)		
Anterior semicircular canal	51 (19.92)	31 (18.90)		
Mixed type	5 (1.95)	4 (2.44)		
DHI score (%)			1.271	0.530
Mild	106 (41.41)	59 (35.98)		
Moderate	120 (46.88)	83 (50.61)		
Severe	30 (11.72)	22 (13.41)		
HADS-A scores			14.186	<0.001
Normal	96 (37.50)	33 (20.12)		
Abnormal	160 (62.50)	131 (79.88)		
HADS-D scores			1.564	0.211
Normal	226 (88.28)	151 (92.07)		
Abnormal	30 (11.72)	13 (7.93)		
PSQI scores			5.890	0.015
Normal	145 (56.64)	73 (44.51)		
Abnormal	111 (43.36)	91 (55.49)		
Serum calcium (mmol/L) (%)			1.314	0.252
Normal	116 (45.31)	65 (39.63)		
Abnormal	140 (54.69)	99 (60.37)		
25(OH)D (ng/ml) (%)			6.127	0.013
Normal	97 (37.89)	43 (26.22)		
Abnormal	159 (62.11)	121 (73.78)		

**Figure 1:**
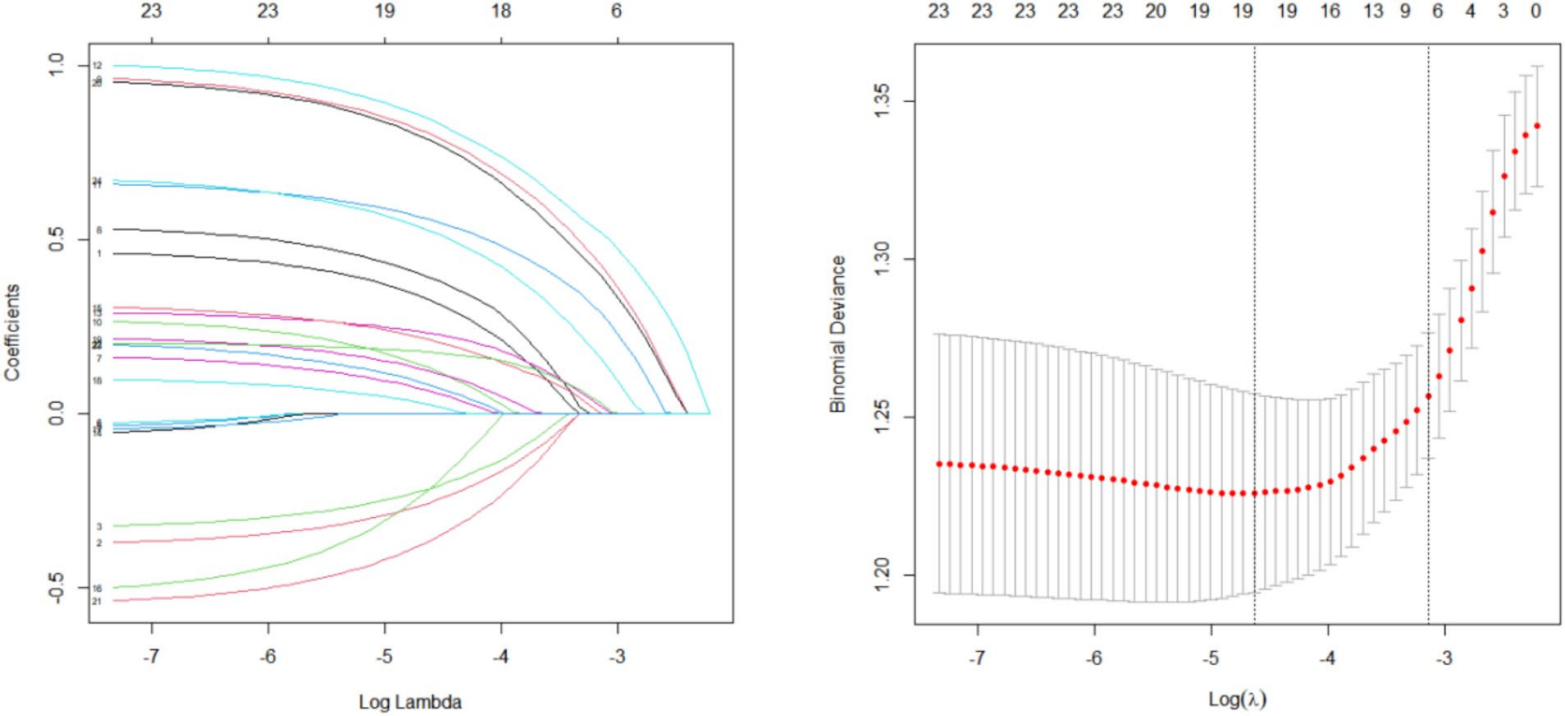
LASSO regression used to screen the predictor variables.

Using LASSO regression, 24 variables were identified as non-zero coefficient predictors. Via 10-fold cross-validation, the optimal *λ* value was selected to reduce the variables’ number while ensuring model fit. Ultimately, lambda.1se was chosen as the best λ value, yielding 7 predictors with non-zero coefficients. These predictors, shown in Figure 1, include hypertension, diabetes, migraine, cervical spondylosis, HDS-A, PSQI, and 25(OH)D.

### 3.3 Multivariate analysis of risk factors

Using recurrence as the dependent variable and seven variables selected through LASSO regression as independent variables, a multivariate logistic regression prediction model was constructed. As shown in Tables 2, 3, hypertension, diabetes, migraine, HADS-A, and 25(OH)D were identified as independent factors influencing recurrence within 1 year after successful reduction in BPPV patients (*p* < 0.05).

**Table 3:** Assignment methods of argument variables.

Variable	Assignment mode
Hypertension	No = 0; Yes = 1
Diabetes	No = 0; Yes = 1
Migraine	No = 0; Yes = 1
Cervical spondylosis	No = 0; Yes = 1
HADS-A scores	Normal = 0; Abnormal = 1
PSQI scores	Normal = 0; Abnormal = 1
25(OH)D	Normal = 0; Abnormal = 1

### 3.4 Nomogram construction and prognostic ability

**Figure 2:**
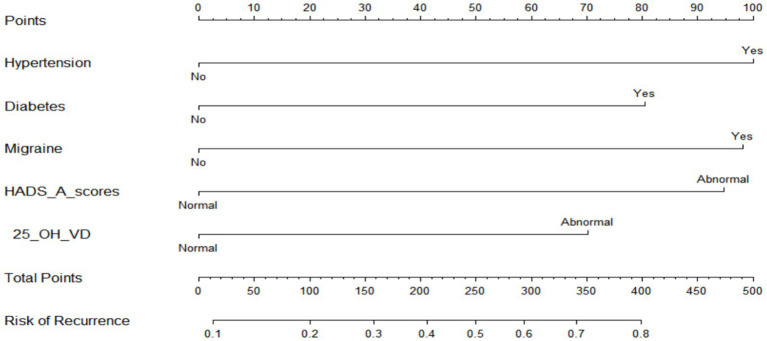
Nomogram prediction model.

**Figure 3:**
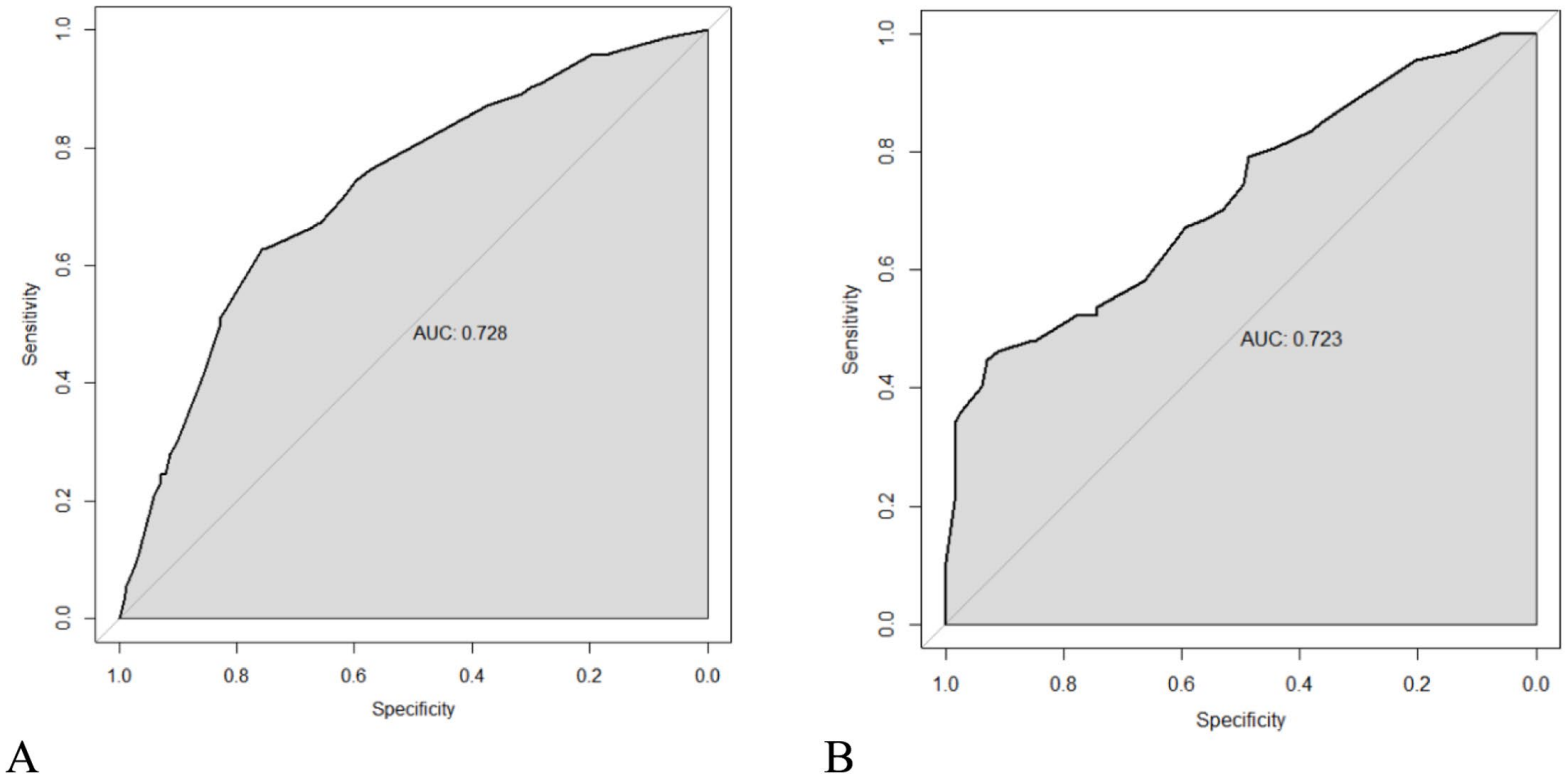
ROC curve of BPPV recurrence risk predicted by the nomogram prediction model. **(A)** Training set. **(B)** Validation set.

**Figure 4:**
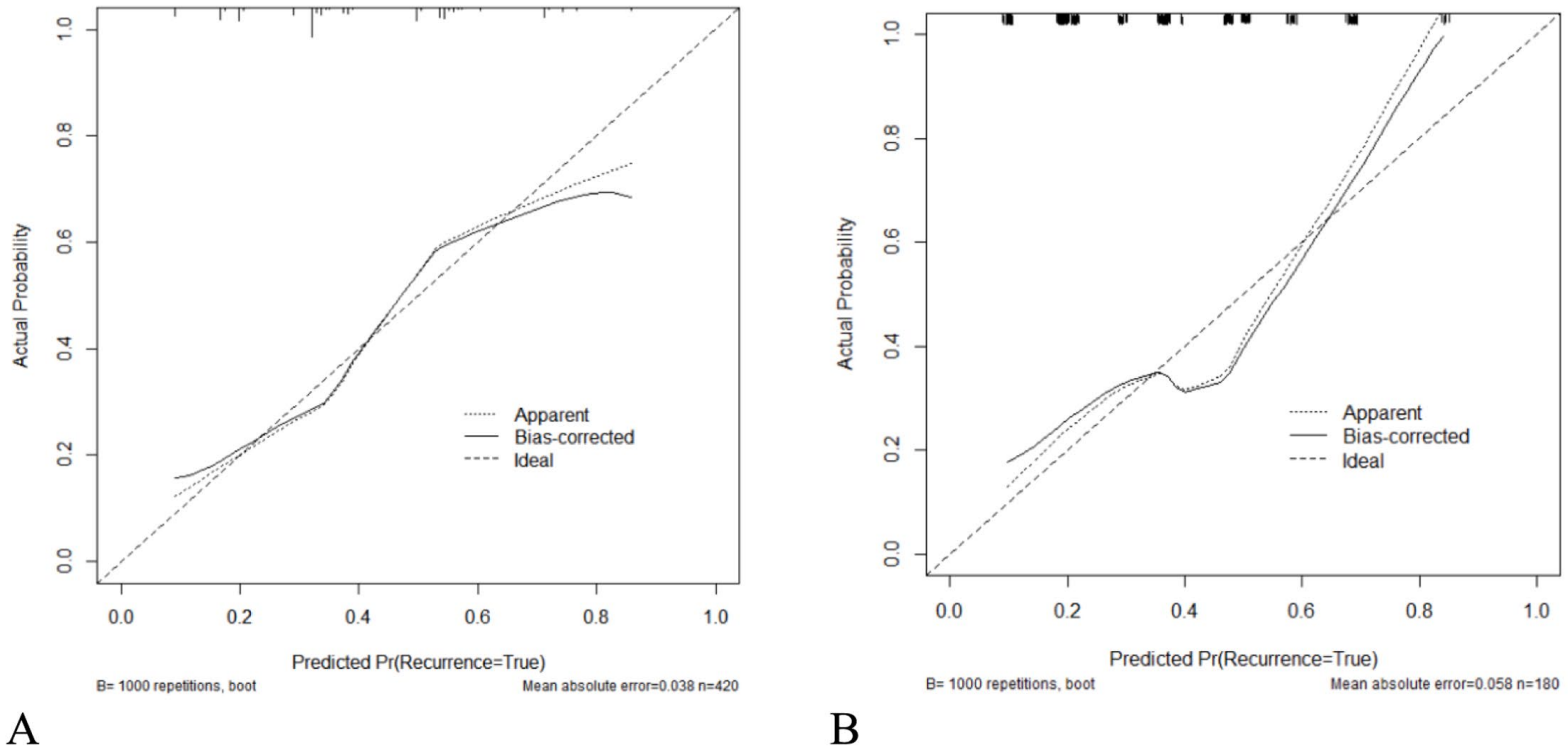
Calibration curve for the prediction model of BPPV recurrence risk. **(A)** Training set. **(B)** Validation set.

As seen in Figure 2, hypertension, diabetes, migraine, HADS-A, and 25(OH)D were used to build a nomogram model for estimating recurrence risk in BPPV patients based on the outcomes of the MLR analysis. The discriminative capacity of this nomogram model was assessed using the ROC curve; for the training set (Figure 3A) the AUC was 0.728 (95%CI: 0.679–0.777); for the validation set (Figure 3B), the AUC was 0.723 (95%CI: 0.645–0.801). With a chi-Square value of 8.710 and *p* = 0.368 for the training set and a chi-Square value of 13.302 and *p* = 0.102 for the validation set, the HLGOF test revealed a satisfactory model fit. Good agreement between expected and actual values was shown by the calibration curve (Figure 4) showing no appreciable departure from the ideal curve (Table 4).

**Table 4:** Multivariate logistic regression analysis of recurrence in BPPV patients.

	*B*	S.E.	Wald	*p*	OR	95%CI
Hypertension	0.906	0.243	13.853	<0.001	2.474	1.535–3.986
Diabetes	0.709	0.233	9.239	0.002	2.031	1.286–3.208
Migraine	0.853	0.237	12.895	<0.001	2.346	1.473–3.736
Cervical spondylosis	0.375	0.221	2.871	0.090	1.455	0.943–2.244
HADS-A scores	0.863	0.25	11.909	0.001	2.371	1.452–3.872
PSQI scores	0.351	0.219	2.568	0.109	1.420	0.925–2.181
25(OH)D	0.628	0.239	6.89	0.009	1.875	1.173–2.997

**Figure 5:**
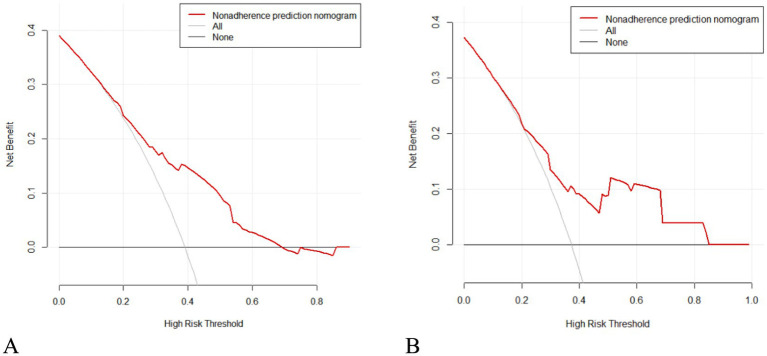
DCA curve of the nomogram model. **(A)** Training set. **(B)** Validation set.

According to the decision curve analysis (DCA), when the high-risk threshold probability is between 0.200 and 0.680 for the training set, and between 0.210 and 0.850 for the validation set, the nomogram model demonstrates high clinical utility for predicting recurrence in BPPV patients. The DCA results are shown in Figure 5.

## 4 Discussion

600 patients were enrolled in this research, with 420 patients randomly allocated to the training set and 180 patients to the validation set at a 7:3 ratio. Within 1 year after manual reduction, 235 cases experienced recurrence, while 365 cases did not, yielding a recurrence rate of 39.17%. In the training set, 164 cases relapsed, 256 did not, with a recurrence rate of 39.05%; in the validation set, 71 cases relapsed, 109 did not, with a recurrence rate of 39.44%. LASSO regression and multivariate logistic regression identified hypertension, hyperglycemia, migraine, HADS-A, and 25(OH)D as independent risk factors for BPPV recurrence after successful reduction.

BPPV recurrence was found to be closely related to hypertension and hyperglycemia. Messina et al. conducted a study on 2,682 BPPV patients, finding a higher incidence of audiological symptoms, such as hearing loss and tinnitus, among BPPV patients with hypertension (2). Similarly, Tan et al. observed that BPPV patients with hypertension required more CRP treatments to achieve successful outcomes compared to those with only hypertension (21). Studies have also indicated that 14% of BPPV patients have diabetes, typically in the 65–74 age group (22). A retrospective analysis of 3,933 individuals by D’Silva et al. found a BPPV prevalence of 46% in type 2 diabetes’ patients compared to 37% in those without (23). This study confirms the relation between BPPV recurrence and both hypertension and hyperglycemia, consistent with previous research findings. Cerebrovascular disease is considered a high-risk factor for BPPV recurrence, with hypertension and diabetes both regarded as vascular risk factors (20). The inner ear’s blood supply is derived from the vertebrobasilar artery system, particularly branches of the anterior inferior cerebellar artery that extend to the vestibular artery (24). Due to its specific anatomical position, the labyrinthine region is susceptible to ischemia. Diabetes may also lead to metabolic acidosis, lowering the pH of endolymphatic fluid and accelerating the breakdown of otolith calcium carbonate (25). Hypertension can induce tissue hypoxia and degeneration in the cochlea and vestibule (26). However, this study did not record specific blood pressure or blood glucose values for patients. Future studies could focus on collecting these values to determine the threshold that influences BPPV recurrence, providing a more precise basis for clinical diagnosis and treatment.

Migraine also emerged as a risk factor for BPPV recurrence. Research has indicated that migraine patients get BPPV twice as often compared to the general population (27). Calhou’s research on 425 migraine patients found a significant relationship between migraine severity and vertigo symptoms (28), while Faralli et al. demonstrated a higher prevalence rate in BPPV patients with migraines (29). Migraine-related recurrent vasospasm or vestibulo-microvascular disorder may cause otolith displacement and inner ear injury into the semicircular canal, triggering BPPV (27). While there is no direct pathophysiological relation, this study revealed that patients with a migraine history face a higher risk of BPPV recurrence. In clinical practice, thorough inquiry into migraine history and relevant examinations are essential for BPPV patients.

The study also indicates a significant relationship between BPPV recurrence and anxiety levels as measured by HADS-A. Depression and anxiety were shown to play crucial roles in dizziness. One study of 870 BPPV patients reported an anxiety prevalence of 47.4% (30). The underlying mechanisms linking BPPV with anxiety and depression remain unclear but may include: (1) vestibular vertigo triggering anxiety symptoms through neural circuits in the parabrachial nucleus; (2) anxiety and depression leading to vasospasm via neuroinflammatory responses, causing BPPV; (3) links between anxiety, depression, and neuroendocrine function, affecting inner ear blood flow. Anxiety and depression interactions with BPPV can lead to adverse outcomes like dizziness and recurrence after CRP (31). This study found that HADS-A is a risk factor for BPPV recurrence, whereas depression (HADS-D) is not. This may be due to the relatively short disease course of 1 year in this study, possibly insufficient to manifest depressive symptoms. Future multi-center studies will incorporate a larger sample and patients with varied relapse durations to further validate these findings.

The study found a strong relation between BPPV recurrence and 25-hydroxyvitamin D deficiency. Kahrman et al. found that 93.5% of BPPV patients had low vitamin D levels (32), and Jeong et al. illustrated that 80% of BPPV patients had vitamin D levels below 20 ng/mL (33). Additionally, Talaat et al. observed a significantly higher prevalence rate in patients with severe vitamin D deficiency (34). This study suggests that 25-hydroxyvitamin D deficiency (levels <20 ng/mL) is a risk factor for BPPV recurrence. The cochlea contains calcium carbonate crystals, and vitamin D receptors in “inner ear epithelial cells” regulate Ca^2+^ binding protein expression. Animal studies have shown that otolith solubility increases as free calcium concentration in cochlear endolymph decreases (35). Vitamin D deficiency, a global health issue, not only impacts calcium ion concentration in vestibular lymph but also affects otolith metabolism, absorption, and regeneration, increasing degeneration and shedding risks (36). Recent research has shown that vitamin D supplementation can help control BPPV recurrence rates (37). Future studies will investigate different intervention strategies for BPPV recurrence risk factors, continuously observing the impact of these factors post-CRP treatment to reduce BPPV recurrence.

The proposed nomogram holds significant potential to optimize clinical decision-making in BPPV management. First, it enables rapid risk stratification at the point of care. By inputting five readily available clinical variables (hypertension status, blood glucose level, migraine history, anxiety score, and 25(OH)D concentration), clinicians can visually quantify a patient’s 1-year recurrence probability through the nomogram’s graphical interface. This empowers targeted interventions: high-risk patients could be prioritized for intensive follow-up and proactive management of modifiable factors—such as vitamin D supplementation for deficient patients or tighter glycemic control in diabetics. Second, the model facilitates personalized prevention strategies. For instance, migraineurs with elevated HADS-A scores might benefit from combined vestibular suppressants and cognitive-behavioral therapy to break the anxiety-vertigo cycle. Conversely, low-risk patients could avoid overtreatment through watchful waiting. Third, at a systemic level, this tool may improve healthcare resource allocation. Emergency departments could integrate the nomogram into electronic health records to flag high-risk BPPV cases during triage, ensuring timely specialist referrals. Additionally, the model’s variables align with routine laboratory panels and comorbidities screening, minimizing implementation costs. Future studies should prospectively validate its impact on reducing recurrence rates and healthcare utilization.

## 5 Limitations

This study predicted the recurrence risk of BPPV patients after successful reduction. It employed variable screening using LASSO regression and subsequently applied the screened variables to logistic regression to construct a predictive model. The model established good differentiation and calibration in both the verification and training sets. However, the recurrence observation period in this study was limited to 1 year, which is relatively short; extending the observation period for future participants would provide more comprehensive insights. Lastly, this study did not involve a prospective cohort analysis of risk factors. Future research could focus on developing prospective intervention strategies to prevent BPPV recurrence based on identified risk factors.

## 6 Conclusion

In summary, a prediction model developed in this study, based on the concentrations of hypertension, hyperglycemia, migraine, HADS-A, and 25(OH)D, is simple, easy to use, and demonstrates good accuracy and predictive efficiency. It can identify high-risk patients with recurrence after successful BPPV reduction in the early stages, from multiple dimensions. This model provides valuable guidance for formulating preventive measures to reduce the risk of recurrence in BPPV patients following treatment.

## Data Availability

The datasets presented in this study can be found in online repositories. The names of the repository/repositories and accession number(s) can be found in the article/supplementary material.
